# 300 mm Large Area Wire Grid Polarizers with 50 nm Half-Pitch by ArF Immersion Lithography

**DOI:** 10.3390/nano12030481

**Published:** 2022-01-29

**Authors:** Jungchul Song, Jae Sub Oh, Min Jun Bak, Il-Suk Kang, Sung Jung Lee, Ga-Won Lee

**Affiliations:** 1 Office of Nano Convergence Technology, National NanoFab Center, Daejeon 34141, Korea; justsong@nnfc.re.kr (J.S.); jsoh@nnfc.re.kr (J.S.O.); mjpark@nnfc.re.kr (M.J.B.); iskang@nnfc.re.kr (I.-S.K.); 2 Division of Electronics Engineering, Chungnam National University, Yusung-gu, Daejeon 34134, Korea; 3 Office of Research and Development, Pavonine Korea, Inc., 33, Hogupo-ro, Namdong-gu, Incheon 21693, Korea; lsj@pavonine.net

**Keywords:** ArF immersion, wire grid polarizer, flare, OPC, LFC, stitching, 50 nm half-pitch

## Abstract

The large area wire grid polarizers (LA-WGPs) with 50 nm half-pitch were fabricated using ArF immersion lithography overcoming the limit of the shot field size. To realize the 50 nm line and space patterns on a 300 mm wafer, a zero-distance stitching process that connects the shot fields is suggested. To compensate for mutual interference between the shot fields which is called the local flare effect (LFE), the shot field arrangement is changed with optical proximity correction (OPC). Using a master wafer produced by the suggested method, 300 mm large-area WGPs were fabricated by the nano-imprint process. The WGPs have more than 80% transmittance in the visible light region, and the possibility of performance improvement can be confirmed depending on the number and method of the etch process.

## 1. Introduction

Wire grid polarizers (WGPs) are receiving considerable interest for broad-band applications such as microdisplay-based projection systems because of their compactness, good polarization efficiency, wide field of view, and long-term stability compared to polymer or thin film-based polarizers such as PVA polarizers [1,2]. In the WGPs, an array of periodic metal wire grating is formed on a transparent substrate. The space between wires must be less than the wavelength of the incident radiation and to cover the entire visible light spectrum, and a pitch of less than 140 nm is required. WGP can replace PVA polarizers used in the industry. Compared to the PVA polarizing plate, the durability, minimal thickness, transmittance, and heat resistance of the WGP are all excellent. The thinner polarizing plate can express a smoother bend when applied to a foldable display. In addition, when applied to the filter of an autonomous vehicle camera, the sun’s reverse reflection light can be blocked to further clarify object recognition. In addition, it can be applied to many industrial fields such as fingerprint recognition, vehicle display, sunglasses, and 3D printing [3]. Despite many advantages, WGP must overcome the limitations of production capacity and size in order to replace PVA [4]. An electron-beam lithography can provide the resolution for the subwavelength metal wire, but its throughput is very low. The argon fluoride excimer immersion (ArFi) scanner to be tested in this paper is being actively used in the semiconductor photolithography process. It has a production throughput suitable for semiconductor production and has a resolution of 80 nm pitch in line and space patterns. However, it also has a size limit of WGP production due to the size of the shot field [5]. To overcome this, multiple single shot fields must be stitched together to create a large area grid. In general, this shot field stitching is not executed perfectly and stitching error is generated periodically with the size of a single shot field requiring precise process control for large-area WGP (LA-WGP). The main cause of error is stray light, accelerating the error in reducing the shot field interval to zero. In the ArFi lithography system, the light passes through a projection lens composed of dozens of quarters even after a mask. In the exposing, the stray light is generated by reflection from the optical element’s surface or by the non-uniformity of the refractive index of the optical materials. This stray light is called flare and exposes unintended areas on a wafer [6] causing mutual interference between the short fields, which is called the local flare effect (LFE). Several techniques for modeling the flare have been reported [7,8]. In the manufacturing of semiconductor die, the scribe widths between the dies should be reduced to lower the fabrication cost. Considering LFE, a 13 μm space is reported to be needed for the LFE in total 96 μm including the dicing, overlay mark and LFE blank area [9].

As previously mentioned, to fabricate a LA-WGP, a stitching process is required with the shot field interval to zero, and the LFE must be minimized. When the shot field interval is removed, the flare effect is repeated once, twice, or up to three times causing an over-dose problem. The over-dose due to interference intensity occurs in the shot boundaries by flare and diffraction, reducing the line critical dimension (CD) to 70% in our experiment. Furthermore, when the pitch is 140 nm or less, the CD variation becomes larger for the same intensity change. When the LFE is repeated twice or more, the interference is so much that not only the pattern but also the entire photo resist (PR) disappears. That is, LFE affects not only the line CD but also the height of the PR, which is sharply reduced in the three iterations of LFE and very vulnerable to reactive ion etching (RIE). Therefore, the LFE must be minimized with the repetition of LFE as much as possible.

In this study, a zero-distance stitching process between the shot fields is proposed for fabricating LA-WGP with 50 nm half-pitch using ArFi lithography with local flare collection (LFC) based on chip array adjustment and mask’s optical proximity collection (OPC). For OPC, it is possible to measure the flare intensity according to the line CD of the PR by adjusting the field distance. The exposure range (shot field) of the mask is 20 mm × 20 mm on a Si-wafer, and the pattern pitch and CD are uniformly formed on an entire 300 mm Si-wafer with the shot field spacing to zero. The transmittance of LA-WGP using ArFi lithography is compared with that of WGP fabricated using E-beam by an interferometer. Fabricating the WGP with ArFi lithography overcomes the throughput limitation of E-beam lithography, and the limitation in shot field size of interferometric exposure.

## 2. Experiment and Simulation

### 2.1. Local Flare Effect

An ArF excimer laser immersion scanner was used to fabricate a 50 nm half-pitch line and space (L/S). The illumination system has the numerical aperture of the lens system, NA of 1.35, Sigma out 0.989, Sigma in 0.910, and a diffraction optical element (DOE) Dipole 35Y. The resist was a chemically amplified positive tone resist with a thickness of 90 nm. Based on the optimized process constant using the illumination system, the limit of ArFi resolution is 38 nm half-pitch based on L/S. 

**Figure 1:**
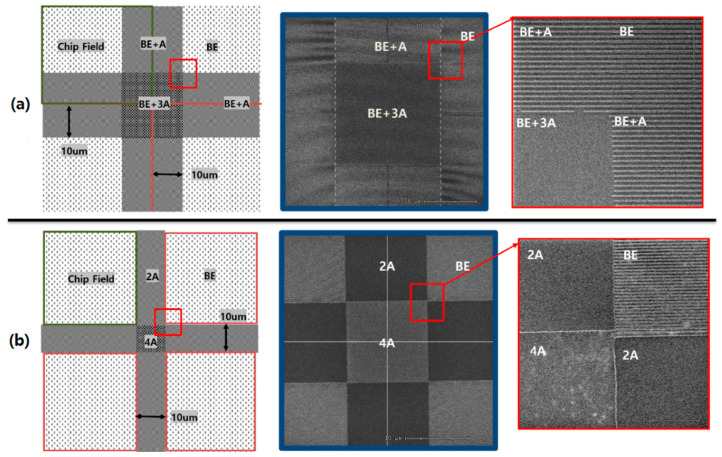
Chip field (shot field) layout for stitching and the photoresist CD according to the number of repetitions of the LFE (**a**) when the shot field interval is set to zero and (**b**) the shot field interval is 10 μm. There are 50 nm patterns of L/S ratio of 1:1 in chip field.

Figure 1 shows chip field (shot field) layout for stitching and the photoresist CD according to the number of repetitions of the LFE. The chip field has a 50 nm pattern with a L/S ratio of 1:1 and LFE occurs up to 10 μm beyond the field and therefore, the distance of the LFE in the section where the two fields meet becomes a total of 20 μm. In the figure, the best energy section (denoted by BE) is where there is no chip field interference, and the ideal 50 nm line CD pattern appears. The section where LFE occurs once is expressed as A, and three times is as 3A. When the shot field interval is set to zero, the line CDs are measured to be 50 nm, 38 nm in BE and BE + A, respectively, but approaching 0 nm in the BE + 3A as shown in Figure 1a. Figure 1b is when the shot field is separated by 10 μm and so the flare effect only exists in the interval. The LFE caused by two fields is expressed as 2A, and an LFE due to four fields is as 4A. 2A and 4A received flare intensities of two and four times, respectively. The PR remained in the 2A section as it is, while the entire PR disappeared in the 4A. Considering that the open threshold intensity of PR is 14 mJ, the experimental results mean that the flare intensity in 2A is smaller than 14 mJ, and in the 4A where the entire PR disappears it is larger than 14 mJ. Therefore, A is greater than 3.5 mJ and less than 7 mJ. The BE of the 50 nm is 24 mJ. The intensity at which the line CD becomes 38 nm due to overdose in the 50 nm target pattern is 30 mJ. Therefore, it can be confirmed that the amount in A is 6 mJ, which is 25% of the BE.

**Figure 2:**
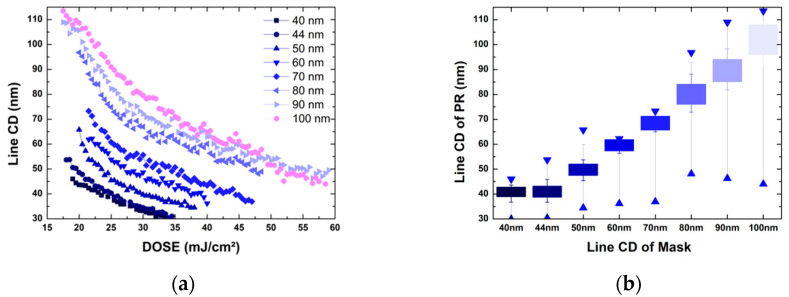
(**a**) Line CD of PR dependence on to intensity (DOSE) in 1:1 L/S Pattern accoridng to line CD of mask. (**b**) Box range is the effective CD variation with ±5% of the target (mask) CD, and stick range is the line standing range.

Figure 2a shows the line CD of PR dependence on the intensity (DOSE) in the 1:1 L/S pattern according to line CD of mask. The larger the mask CD, the PR CD can stand in response to increased intensity. When LFE of 6 mJ is interfered three times on 100 nm line CD, the intensity of 18 mJ is added. When the intensity in BE is 25 mJ, the total energy becomes 43 mJ in BE + 3A, and line CD of PR is reduced to 70 nm, but it is still standing. However, in the 50 nm line CD, there is no PR when LFE interferes 3 times.

In Figure 2b, the box shows the CD range where the valid values of ±5% in 1:1 L/S pattern from 40 nm to 100 nm mask CD, and the range where the CD bar stands is marked with a stick. The smaller the mask CD, the smaller the range it can stand. This means that the smaller the line CD, the greater the influence of the LFE. In the LFE section, the 100 nm line CD is reduced to 70 nm, and the 50 nm line CD has no PR. As the CD becomes smaller in the stitching process, the CD variation due to flare becomes larger. In addition, the effective box range of ±5% is also reduced, so precise control of the energy margin for the target CD is required.

### 2.2. Local Flare Collection

**Figure 3:**
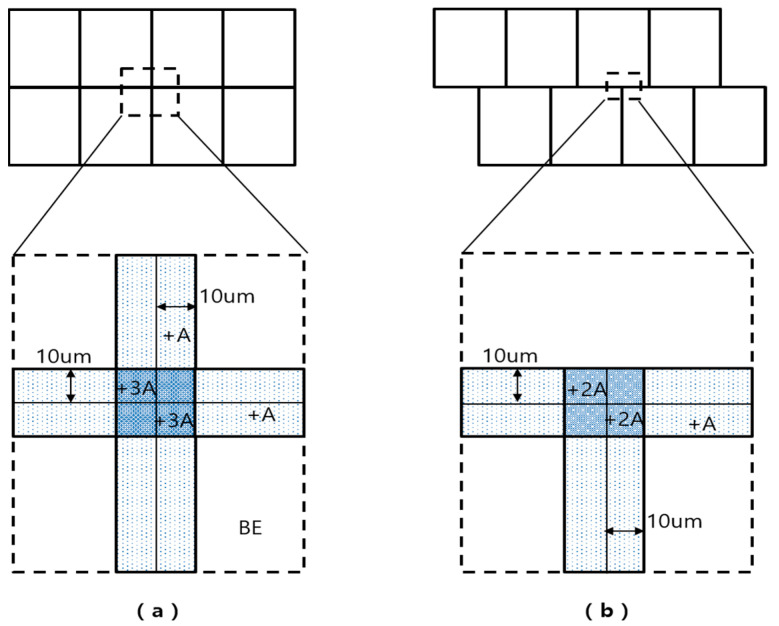
LFE according to the shot field arrangement. Array in which (**a**) the row is not shifted and (**b**) is shifted by 20 μm or more.

When the shot field array is arranged as a matrix and the interval is set to zero (wafer chip size and mask shot size are same), the maximum flare repetition can be three times (+3A) as shown in Figure 3a. In this experiment, the short fields are shifted more than 20 μm to the next row as in Figure 3b. The repetition number is reduced, and the maximum flare repetition becomes two times (+2A). The reduction in the maximum number of flare repetition can result in a smaller CD width realization of the stitching process. As demonstrated earlier, the amount of overdose due to one interference becomes 125%, which is 25% higher than that of the BE. It increases proportionally as the LFE is repeated. This is because each shot exposes the same energy and passes through the ArF lithography’s projection lens, which causes the same stray light. Two flare repetitions are exposed to 150% intensity and three flare repetitions to an intensity of 175%. At 1:1 L/S 50 nm, BE is 24 mJ, +A is 30 mJ, +2A is 36 mJ, +3A is 42 mJ. When processed by Figure 3b array, the maximum intensity in LFE becomes 36 mJ (+2A), and it can stand from 50 nm line CD to 38 nm line CD as shown in Figure 2a.

For the superior local flare collection (LFC), OPC is also applied. As the LFE repetition is reduced by changing the shot field arrangement, the CD variation can decrease. The reduced CD variation can increase the efficiency of OPC in the stitching process. The resolution of the ArFi scanner RES is k_1_·λ/NA, where λ is wavelength of illuminating light and k_1_ is process constant which depends on the photoresist sensitivity, the photomask complexity, and the lift-off conditions. The maximum resolution of ArFi by increasing the k_1_ to the maximum (using a phase shift mask and a dipole DOE) is 38 nm. This means ArFi cannot form patterns in half-pitch patterns below 38 nm. Either the NA should be larger, or the light wavelength should be reduced as EUV. For this reason, there is little synergistic effect to apply OPC in less than 38 nm based on line and space [10]. 

The pitch and line CD should be uniform in the stitching process. The line CD of PR can be controlled by adjusting the ratio of line and space area and the transmitted intensity [11]. Overdosing the transmittance reduces the line CD as the energy reflected on the lower film reaches the side of the PR [12]. However, PR activation by reflection intensity increases at the lower end and causes line CD slop or collapse. For this reason, there is a limit to the CD that can be reduced by overdose, and it can be expressed as a section where the line CD can stand without falling. It can be seen from Figure 2b that the CD standing section increases as the CD becomes larger. Depending on the line and space ratio, DOSE shifts at a large rate, but it can be seen from Figure 3a that the CD standing range does not differ significantly. An increase in the CD standing area has the same meaning as an increase in the range to which OPC is applied [13].

**Figure 4:**
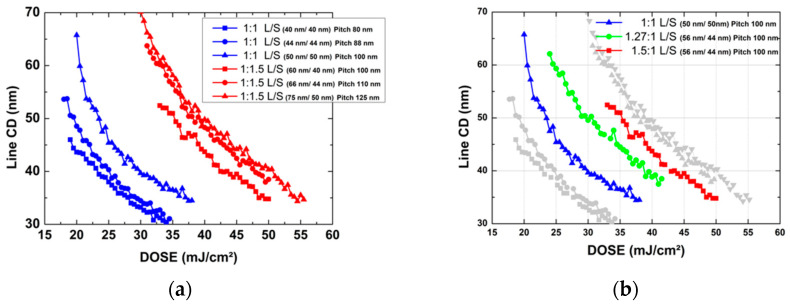
Line CD dependence on DOSE (**a**) according to Mask open ratio and (**b**) according to L/S ratio applicable to +A and +2A sections at the same pitch (line 50 nm).

Before applying OPC for local flare collection, the CD dependence for best energy according to the L/S ratio in Figure 4a was tested. At 1:1 L/S with Space (Mask open ratio 0.5) 50 nm, (BE) Intensity is 23 mJ, and 1.5:1 L/S with Space (Mask open ratio 0.4) 50 nm increases to 39 mJ. BE shows that 170% of the energy point shifts with the change in mask open ratio. Depending on the Mask Open ratio, the intensity (DOSE) of all 40 nm, 44 nm, and 50 nm (Space) increase at the same rate. Using this, the interference intensity caused by flare can be compensated by changing the mask open ratio in the stitching process with the same pitch. At 50 nm stitching, 125% increased 30 mJ is transmitted to BE (24 mJ) in the BE + A section, and when 1.27:1 L/S Space 44 nm (mask open ratio 0.44) is applied, the pitch is 100 nm and a 50 nm line CD can be processed. Using the same principle, if 1.5:1 L/S Space 40 nm (mask open ratio 0.4) is applied to the area where 36 mJ, which is increased by 150%, is transmitted in the BE + 2A section, the 50 nm line CD can be processed. In addition to the PR used in the experiment, even when other PRs with sensitivity are used, this method can induce OPCs capable of performing the stitching process.

If the shot field array is used as the basic matrix structure, +3A section occurs, and it is necessary to find a region where 50 nm is expressed at 175% of energy (42 mJ) compared to BE. The mask open ratio should be further reduced, but the maximum resolution for ArFi lithography is 38 nm, which is impossible. If CD is reduced further, the light will not be transmitted. The reason why 76 nm, which is the ArFi minimum pitch, is not used for large-area WGP production is that the space CD must be reduced as compensation for LFE, but an ArFi resolution of 38 nm or less cannot be used. In order to perform the 50 nm stitching process, both the row shifting shot field array and OPC must be applied to fabricate using ArFi lithography.

## 3. Results and Discussion

### 3.1. LFC Result

**Figure 5:**
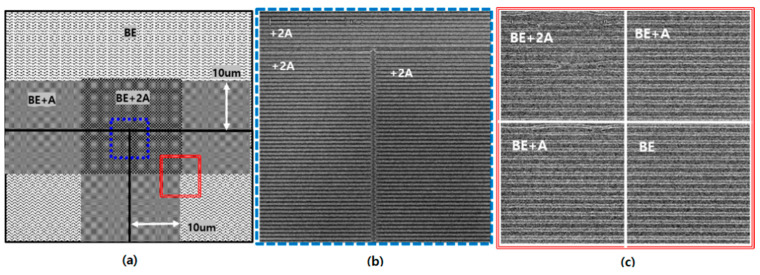
(**a**) Schematic of shot field boundary after OPC. CD_SEM (Scanning Electron Microscope) image of (**b**) the blue dotted line and (**c**) the red solid line.

Figure 5 is a scanning electron microscope (SEM) picture showing the top CD view according to the number of LFE repetitions after changing the shot field array with mask OPC based on the previous demonstration. A line CD of 50 nm is maintained at 100 nm pitch in all sections BE, +A, and +2A, and it can be seen in the top view CD that a 50 nm was processed throughout the LFE section.

**Figure 6:**
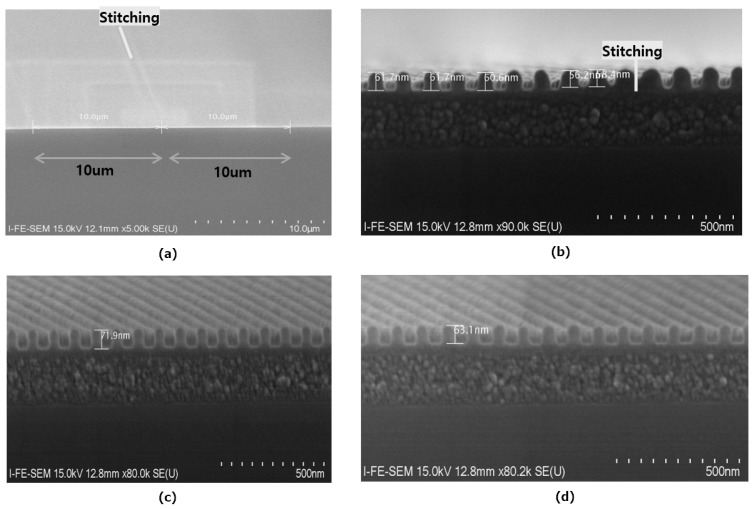

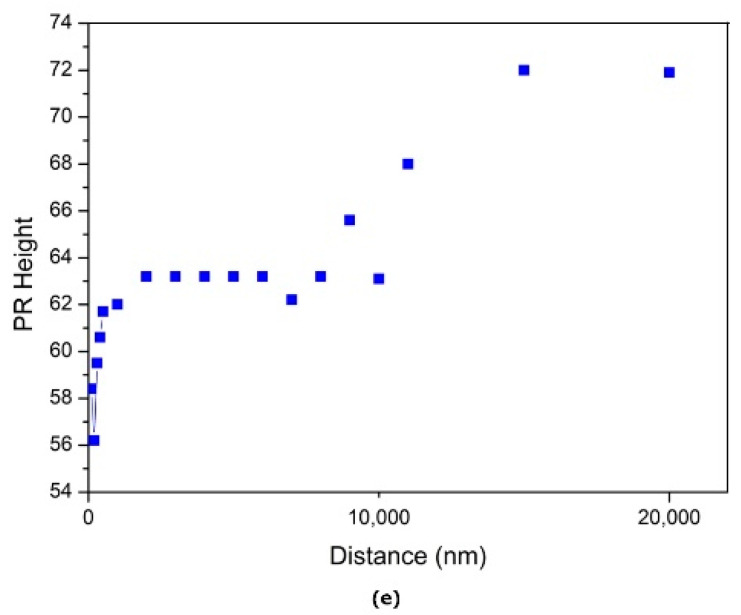
Cross-section image of PR according to the distance of the shot field (PR height difference due to flare intensity), (**a**) Stitching cross-section SEM, (**b**) LFE 0~500 n distance section, (**c**) normal PR height, (**d**) LFE 9 μm distance section, and (**e**) PR height according to LFE distance.

Figure 6 shows the cross-sectional SEM picture of the LFE area and the PR height according to the distance of the LFE. The boundary of the LFE cross section was checked using field emission scanning electron microscopy (FE-SEM) and the height of the PR was found to change. Flare intensity depends on the density and distance of the pattern. The closer to the density pattern (shot field), the more the background light (flare effect) is affected by additional stray light from the lithography [14]. The CD variation in the top view is not valid despite receiving different flare intensities according to distance within the LFE. In the process to which OPC was applied, the top view CD did not change depending on the LFE, but when checking the cross section, it was confirmed that the height of the PR was changed. The height of the PR before the exposure is 90 nm and the loss due to pattern exposure is 10 nm. The average height of the BE section is 85 nm. In the +A section, the height of the farthest PR from the field boundary is 70 nm, and the closest PR height is 60 nm, resulting in a height variation of 10 nm. The intensity difference due to distance in LFE causes a relatively large loss in PR height as the pitch decreases. According to the aforementioned resolution formula (RES = k_1_·λ/NA), NA should be increased and λ should be reduced for small CD. These changes affect the Depth of focus (DOF). The formula of DOF is DOF = k_2_·λ/(NA)^2^, and as NA increases, DOF decreases further. Similar to Resolution’s formula, k_2_ is the process constant, λ is the wavelength of illuminating light, and NA is the numerical aperture. A small DOF is an important reason to keep the PR height low. The smaller the pitch, the lower the PR can be, and a large proportion of the PR height is lost in the flare effect. The PR height loss caused by the LFE reduces the PR margin in the etch process, which leads to the limitation of the etch depth.

**Figure 7:**
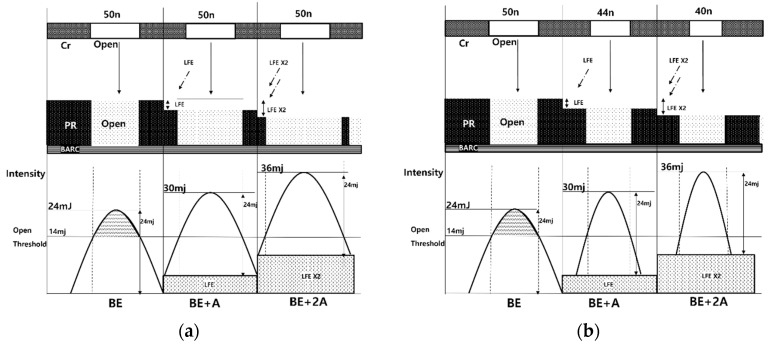
Intensity (DOSE + Flare) simulation of PR activation and PR activation according to the mask open ratio, (**a**) without OPC and (**b**) with OPC.

Figure 7 shows schematically how the light source of ArFi is exposed to PR after passing through the Cr mask. When the same amount of light is exposed, a section where PR responds according to the number of repetitions of LFE was drawn. Since there are dozens of quartz elements between the Cr mask and the PR, there will inevitably be stray light. At this time, stray light is generated by reflection from the surface of the optical quartz element or by the non-uniformity of the refractive index of the optical material [15]. The resulting stray light creates a section that forms the LFE. Reduce the PR height and reduce the bar CD by inducing overdose and exposing the side of the PR. The open threshold of PR is 14 mJ and the best energy of 50 nm pattern is 24 mJ. As the amount of interference caused by stray light (LFE repetition) increases, more sections exceed the open threshold, which broadens the response area of the PR and induces the entire PR to disappear. By applying OPC to the same pitch, the open threshold section can be made the same even in the LFE section.

As shown in Figure 7b, the width of the transmitted intensity is narrowed by reducing the mask open ratio. Line CD widths beyond the open threshold at the same intensity are made equal. It is possible to fabricate large-area WGP molds by stitching the same line CD. 

### 3.2. Large-Area WGP

WGPs consist of subwavelength periodic metal lines [16,17,18]. The transverse electric (TE) field running along the direction of a metal wire induces free electrons to move along the wire because light is composed of electromagnetic fields. As a result, the polarized light is reflected, similar to light reflecting from a metal surface. However, electrons cannot traverse very far between metal wires when incident light is used to apply an electric field perpendicular to the wires, so a transverse magnetic (TM) field is transmitted through the WGPs [19]. The spectral working range and the optical properties of a WGP (such as transmittance and extinction ratio) are determined by the grating material and the structural parameters of the metal grating such as the period, grating height, and linewidth. In the last two decades various research results on the fabrication of WGPs using nanoimprint lithography have been reported [20,21]. 

The stitching pattern on the front surface of the Wafer made based on the method provided in this paper becomes Si-master. Si-master can be used to fabricate film-based polarizers through nanoimprint lithography. The nanoimprint lithography principle is quite simple. Nanofeatures are defined on the surface of a hard mold, which is then used to emboss a polymer cast onto a wafer substrate under controlled temperature and pressure [22]. Si-master’s 50 nm stretching pattern becomes Nanofeature. The nanoimprint lithography process is carried out with a roll-to-roll process on PET film and imprinted on UV resin. PET film with the same pattern as Si-master is refined by plasma etching (Ion Milling). After that, aluminum (Al) is deposited obliquely on the made pattern. For gradient deposition, the loading chuck is processed with an angle. By depositing at an angle, the thickness of Al can be made thin and high. The deposited Al has a height of 128 nm, a line width of 52 nm, and a pitch of 100 nm.

**Figure 8:**
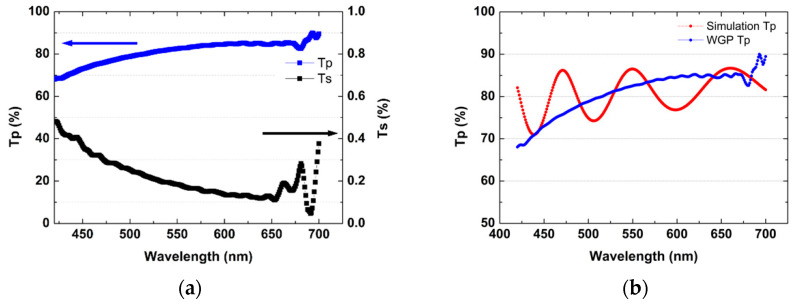
(**a**) WGP Tp, Ts and (**b**) Measurement transmittance of Simulation and WGP.

A polarimeter with a halogen light source was used to characterize the polarization performance of the 300 mm wafer size WGP. The light source passes through the PVA polarizing plate and then passes through the LA-WGP disposed parallel or vertically. Through this, the polarization transmittance of LA-WGP passing PVA was measured using a spectrophotometer (CL-500A). The measurement conditions of the darkroom were 0.5 lux or less. The value from the light source when the PVA and WGP arrays cross vertically is called Ts (s-polarized transmittance), and when the PVA and WGP arrays are equally parallel, the polarized light is called Tp (P-polarized transmittance). The data on the transmittance of the manufactured WGP are summarized in Table 1. The contrast ratio (CR) is the ration of Tp and Ts. When the visible light region is represented in units of wavelength 1 nm of the light source, graphs of Tp and Ts are shown as in Figure 8a. Figure 8b is a comparison of the actual transmittance of a laser passing through the Al pattern (width 52 nm, pitch 100 nm, height 128 nm) wire grid on the fabricated large area WGP, obtained by experiment, with the virtual transmittance data using a program called G-solver. 

The G-solver program does not consider an Al gradient deposition, but the virtual result uses the Maxwell equation for an entire Al Line patterned on an infinite plane. In the manufactured WGP, the entire line is not Al because Al is deposited obliquely on the PET film. In addition, the virtual value of the simulated Sim Tp in the form of a wave uses a Laser light source (LED), while the Tp value of the produced WGP is from a halogen light source. There is a difference in amplitude in the resulting values, due to the difference between the light sources and the Al pattern shapes. Considering these two points, it can be confirmed that the transmittance of the prepared WGP is not different between simulation data and experimental data. 

The transmittance of the 300 mm wafer WGP in the visible light region was the same transmittance (80 to 90%) as reported for other WGP (line width of 100 nm or less) manufacturing experiments. The WGP transmittance depends on the height as well as the width of the Al pattern [23]. Previously, the PR height was reduced to 600 Å due to repeated exposure of flares in the process of manufacturing standard molds before nanoimprint lithography.

**Figure 9:**
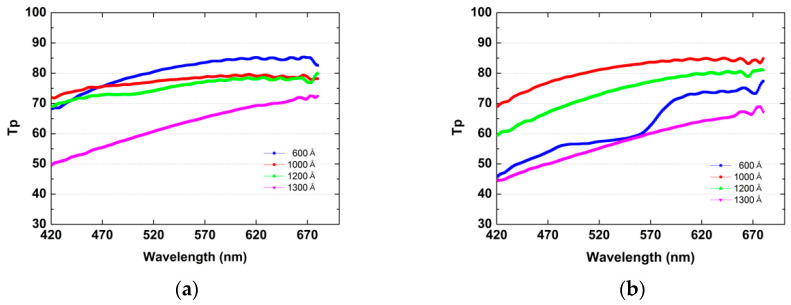
Comparison of transmittance according to Master mold pattern depth. (**a**) Ion Milling etch once (**b**) Ion Milling etch twice.

The maximum master mold etch depth is approximately 1500 Å due to the limit of the height (600 Å) of PR in LFE. The pattern height of the master mold is almost etch depth. The Al height of the WGP is determined by the pattern height of the master mold. Prior to the nanoimprint lithography process, the depths of the master mold were prepared as 600 Å, 1000 Å, 1200 Å, and 1300 Å, respectively. The PET film-type pattern is completed through nanoimprint lithography process using a master mold with four different heights. Then, plasma etching is further performed to refine the pattern after the nanoimprint lithography. Plasma etching (Ion Milling) was performed once in Figure 9a, and 600 Å showed the highest transmittance. Figure 9b shows the highest transmittance of 1000 Å when plasma etching was performed twice. As shown in the figure, the results of transmittance vary depending on the change of the two parameters of the plasma etching after nanoimprint lithography and the master mold etch depth before nanoimprint lithography.

**Figure 10:**
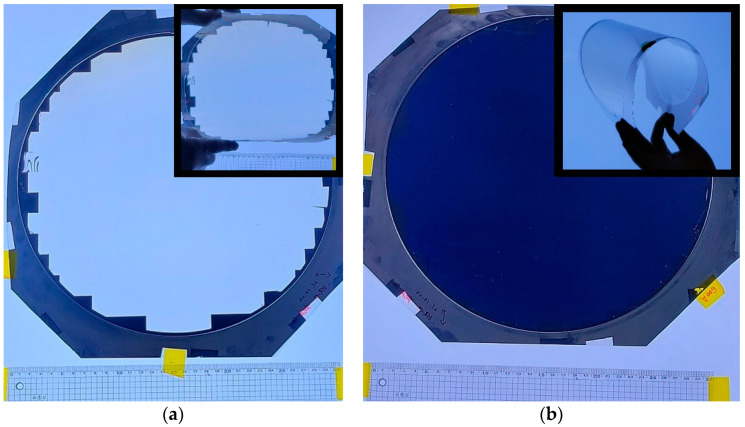
A 300 mm wafer size LA-WGP. (**a**) Tp (PVA film and WGP arranged in parallel) and (**b**) Ts (PVA film and WGP placed vertically). The inlet picture shows that the fabricated LA-WGP is flexible.

The transmittance changes according to the pattern depth of the 300 mm master mold and the RIE process conditions after the nanoimprint lithography process. The transmittance may vary depending on the degree of process optimization. In addition, the transmittance fluctuated depending on the roughness of the line pattern (Al) of the WGP [24]. The transmittance performance can be improved using a process of gradient Al deposition. Figure 10 shows a visual confirmation of Tm polarization and Ts polarization for the manufactured 50 nm large area WGP on a 300 mm wafer size (mold depth 60 nm, ion milling once, Al gradient deposition).

The LA-WGP was placed in parallel or vertically on a large LED light covered with a PVA polarizer to photograph the light passing through the two polarizers (PVA, WGP). The boundary line of the shot field was not visible, nor was the shot uniformity (Mura) caused by CD variation found.

## 4. Conclusions

The advantages of the WGP polarizer over the PVA polarizer are thin thickness, physical durability, high temperature resistance and reflective polarization performance. However, there are limitations in the size of the shot field of lithography and the throughput of E-beam. In this study, the interference between shot fields is reduced from three times to two times by shifting the upper and lower rows of the shot field array. In addition, CD variation was eliminated by applying OPC to the LFE section to compensate for the reduced interference of two flares. With these two methods, the stitching process in ArFi lithography produces an LA-WGP master mold with a size of 300 mm wafer. The overall size of the mold is a 300 mm full wafer, which exceeds the limit of the conventional shot field size (25 mm × 33 mm). After imprinting UV resin on the 300 mm Si master, it is possible to produce LA-WGP with excellent polarization quality and a thickness of 5 μm or less by plasma etching process and oblique deposition of Al. This experiment shows that many industrial products used as PVA polarizers can be applied to WGP. When applied to a foldable display, it will become thinner and the surface of the curved section will be further improved. When applied as a polarizing filter for autonomous vehicles and object recognition cameras, the uncertainty of object recognition due to reflected light is significantly reduced. It can be applied to many industrial fields, such as AR and VR polarizers, fingerprint recognition sensors, position sensors, vehicle displays, and polarized sunglasses. This study also demonstrated that the performance of LA-WGP can be further improved by optimizing the plasma etching process performed after the PR height of the master mold.

## Figures and Tables

**Table 1:** Transmittance data of WGP in the visible light region RGB. The Contrast Ratio (CR) is the ration of Tp and Ts (Tp/Ts).

450 nm (420~500)			550 nm (500~590)			650 nm (610~680)		
Tp (%)	Ts (%)	CR	Tp (%)	Ts (%)	CR	Tp (%)	Ts (%)	CR
73.0	0.37	199	82.52	0.18	448	84.68	0.12	699

## Data Availability

Data is contained within the article.
